# A daily diary study of self-compassion and adaptive coping behaviours in women with symptoms of bulimia nervosa

**DOI:** 10.1186/s40337-023-00755-6

**Published:** 2023-03-27

**Authors:** Aleece Katan, Allison C. Kelly

**Affiliations:** Department of Psychology, University of Waterloo, 200 University Ave. West, Waterloo, ON N2L 3G1 Canada

**Keywords:** Bulimia nervosa, Daily diary, Multilevel modeling, Self-compassion, Mental health, Adaptive coping

## Abstract

**Background:**

Mental health is more than the absence of illness and includes the ability to cope adaptively with stress. To shed light on the factors that promote mental health in people with eating disorders, this daily diary study examined whether daily and trait levels of self-compassion predict adaptive coping behaviours in women with symptoms of bulimia nervosa (BN).

**Methods:**

Women (*N* = 124) who met the DSM-5 criteria for BN completed 2 weeks of nightly measures assessing their daily level of self-compassion and their daily adaptive coping behaviours, namely, their use of problem-solving strategies, seeking and receiving of instrumental social support, and seeking and receiving of emotional social support.

**Results:**

Multilevel modelling revealed that on days when self-compassion levels were higher than their personal mean level or than the preceding day’s level, participants reported greater use of problem-solving strategies, greater seeking and receiving of instrumental social support, and greater receiving of emotional social support. Daily levels of self-compassion, but not increased self-compassion from the preceding day, were associated with emotional support sought. Further, higher trait self-compassion, as measured by participants’ mean level of self-compassion over the 2 weeks, was associated with increased seeking and receiving of instrumental and emotional social support but not with problem-solving strategies. All models controlled for participants’ daily and mean eating pathology over the 2 weeks, highlighting the unique contribution of self-compassion to adaptive coping behaviours.

**Conclusions:**

Results suggest that self-compassion may help individuals with symptoms of BN cope with challenges in their daily life more adaptively, an integral component of positive mental health. The present study is among the first to suggest that the benefits of self-compassion for individuals with eating disorder symptoms may lie not only in facilitating reduced eating pathology, as evidenced by prior research, but also in promoting positive mental health. More broadly, findings underscore the potential value of interventions designed to build self-compassion in individuals with eating disorder symptoms.

## Abstract (summary)

There is a call in the eating disorder field to identify factors that not only reduce eating disorder symptoms, but also promote positive mental health. Yet, the factors that interventions might target to accomplish both of these goals remain relatively unknown. Self-compassion, which is a way of responding to personal distress with care and understanding, has been linked to fewer eating disorder symptoms. In non-eating disorder populations, self-compassion also promotes adaptive coping behaviours, including problem-solving and calling on others for support, key aspects of mental health. To test whether these benefits of self-compassion extend to people with eating disorders, we had women with symptoms of bulimia nervosa complete questionnaires about their daily level of self-compassion and their daily adaptive coping behaviours every night for 2 weeks. We found that on days when women were more self-compassionate than usual or more self-compassionate than the day before, they reported using more adaptive coping strategies. Further, individuals who were generally more self-compassionate than others over the 2 weeks reported receiving more social support from others. Our results are the first to suggest that, by treating themselves with compassion, individuals with eating disorder symptoms may cope more effectively with challenges in daily life.

## Background

There is a call in the eating disorders field to identify factors that not only reduce eating disorder symptoms but that also bolster mental health. The World Health Organization defines mental health as “a state of well-being in which an individual realizes [their] own abilities, can cope with the normal stresses of life, can work productively and is able to make a contribution to [their] community” [1] (para. 1). At present, there is a substantive body of literature on people with eating disorders exploring factors and treatment approaches that reduce eating pathology; however, there is a dearth of research on factors that promote mental health in this population.

### An introduction to adaptive coping

Although there are various components of mental health, the ability to cope adaptively with stress is particularly relevant to individuals with eating disorders [2, 3]. Coping is a multidimensional construct that refers to the cognitive and behavioural efforts an individual employs to manage internal and/or external demands that they perceive to be distressing [4]. Problem-focused coping refers to efforts that are aimed at eliminating or mitigating the impact of the stressor itself; these efforts can include planning, suppressing competing demands, and/or asking another person for practical or instrumental support [5, 6]. Emotion-focused coping broadly refers to efforts that are aimed at reducing or managing the distress that is triggered by the stressor [5, 6]. Emotional *avoidance* coping strategies involve directing attention *away* from the stressor, for example, via denial or distraction, whereas emotional *approach* coping strategies involve active engagement with one’s emotions, for example, by letting oneself cry or seeking emotional support from others [7]. Whereas emotional avoidance coping is almost always maladaptive and linked to psychopathology, problem-focused and emotional approach coping strategies are regarded as adaptive and related to reduced distress, greater positive mental health, and greater resilience [8, 9].

There is an expansive body of literature suggesting that individuals with eating disorder symptoms experience high levels of daily distress [10, 11], with which they generally cope through emotional avoidance, compromising their mental health [3, 12–14].

### Self-compassion and adaptive coping

Self-compassion is one factor that may promote more adaptive forms of coping in individuals with eating disorders. Self-compassion is conceptualized as a motivation to both attend to one’s suffering from a caring orientation as well as to alleviate and prevent current and future suffering [15, 16]. According to Neff [16], self-compassion involves approaching personally distressing experiences with a mindful and curious stance, a spirit of inner-kindness and warmth, and the perspective that suffering is shared by all humans. Research suggests that self-compassion levels vary between people, with certain individuals reporting higher trait levels than others [17], and also within a person, with a given individual responding to their distress more self-compassionately on some days compared to other days [18, 19]. In individuals with eating disorders, both daily [20, 21] and trait [22, 23] self-compassion levels have been consistently linked to reduced eating pathology, and self-compassion interventions have been found to reduce eating disorder symptoms over time [23–25]. Research has yet to explore whether self-compassion might also promote mental health, and specifically adaptive coping, in this population.

There is theoretical and empirical reason to believe that self-compassion might promote the use of adaptive coping behaviours in individuals with eating disorders. Self-compassion involves being sensitive to one’s distress with an eye toward alleviating it. As a result, self-compassion is likely to motivate individuals to experience and make sense of their feelings, and to enact appropriate strategies to help themselves feel better (e.g., reaching out to trusted others and/or problem-solving) [26]. The empirical association between trait self-compassion and adaptive coping behaviours has been extensively documented outside of the field of eating disorders [27–31] with some evidence suggesting that daily levels of self-compassion are similarly linked to adaptive coping [32, 33]. Thus, it seems likely that self-compassion might also facilitate adaptive coping in people with eating disorders.

### The present study

Using data from a 2-week daily diary, the present study examined whether differences in (1) average, or trait, self-compassion levels between people and (2) self-compassion levels within a person from one day to the next contribute to variability in adaptive coping behaviours in women with symptoms of bulimia nervosa. We hypothesized that there would be a greater use of problem-focused coping strategies (i.e., problem-solving, seeking and receiving instrumental support) and emotional approach coping strategies (i.e., seeking and receiving emotional support) (1) among individuals with higher trait self-compassion levels, and (2) on days when individuals were more self-compassionate than (2a) their personal mean level and (2b) the preceding day. Given that participants’ eating disorder symptoms may influence their choice of coping strategies [34] and their likelihood of being self-compassionate [20], we sought to examine whether the hypothesized associations would emerge even when controlling for participants’ average and daily eating disorder symptoms.

## Methods

### Study data

This study utilized participant data that was previously analyzed by Katan and Kelly [20]. While the present research questions and hypotheses are unique from those in the prior paper and were devised a priori during study development, we acknowledge that the current study was not preregistered.

### Participants

Participants were 124 cis women (*M*_age_ = 26.47, *SD*_age_ = 8.31) who were recruited from online and poster advertisements in the community and from an undergraduate participant pool as part of a larger study. To be eligible, participants had to meet the following criteria: female, 18 years of age or older, binge eating and compensation for binges (e.g., vomit, use laxatives) on average at least once per week, minimum body mass index (BMI) of 18.5 kg/m^2^ (*M*_BMI_ = 26.75, *SD*_BMI_ = 5.46), and nightly internet access.

Interested individuals consented to the pre-screen survey and completed the Eating Disorder Diagnostic Scale [35, 36] via Qualtrics; a trained research assistant then verified symptoms via telephone to ensure they met DSM-5 criteria for bulimia nervosa. All participants provided informed consent prior to study participation. Reimbursement for study participation was an Amazon.ca gift card (up to $50 CAN) and/or study participant pool credit (for some undergraduate participants). The institutional research ethics board approved this study, and the study was conducted in accordance with the institution guidelines and the Tri-Council Policy Statement: Ethical Conduct for Research Involving Humans.

Approximately 33% of the sample reported previously receiving a formal eating disorder diagnosis, and 79% of participants self-identified as having an eating disorder at the start of the study. Further, 9.7% (*n* = 12) of participants reported being in eating disorder treatment at the time of participation; this included outpatient treatment, private therapy, and/or sessions with a dietician. The ethnic composition of the final sample was 46.0% White, 12.1% Chinese, 12.9% South Asian, 7.3% Latin American, 5.6% Black, 5.6% Southeast Asian, 1.6% Filipino, 1.6% Arab, 1.6% Korean, and 5.6% Other.

### Procedure

Participants were asked to complete an online questionnaire about their daily experiences and eating behaviours via Qualtrics for 14 consecutive nights. A link to the questionnaire was emailed to participants at 8:00 p.m. each night of their study participation. All participants were instructed to complete the questionnaire before bed to ensure that all daily eating behaviours were reflected in their nightly reports.

### Measures

The following measures were completed on a nightly basis, each of which was modified for daily use by instructing participants to respond to each measure based on “today.” Omega values were computed for each multi-item measure as an estimate of reliability at the within-persons (ω_w_) and between-persons (ω_B_) level [37].

#### Self-compassion

Daily self-compassion was assessed using the 12-item Self-Compassion Scale-Short Form [38]. Participants used a five-point scale ranging from 1 (*almost never*) to 5 (*almost always*) to indicate how often they behaved in the stated manner that day (e.g., “When I was going through a very hard time, I gave myself the caring and tenderness I needed”). This measure demonstrated an acceptable degree of internal consistency (ω_w_ = .76; ω_B_ = .92).

#### Problem-solving

The 11-item, three-point (1 = *not at all* to 3 = *a lot*) Problem-Solving Subscale from the Coping Strategies Indicator [39] was used to assess the extent to which participants engaged in problem-focused coping strategies (e.g., planning) to manage a situational stressor they encountered that day (e.g., “Rearranged things so your problem could be solved”). This measure demonstrated a high degree of internal consistency (ω_w_ = .90; ω_B_ = .98). This measure was added shortly after data collection began and thus was not completed by 11/124 participants.

#### Instrumental social support

*Instrumental social support sought.* A four-item subscale from the COPE inventory [6] was used to assess social support sought for instrumental reasons. Participants used a four-point scale ranging from 1 (*I didn’t do this at all*) to 4 (*I did this a lot*) to rate the extent to which they sought instrumental social support in response to difficult or stressful events that day (e.g., “I talked to someone who could do something concrete about the problem”). This measure demonstrated a high degree of internal consistency (ω_w_ = .87; ω_B_ = .98).

*Instrumental social support received*. Received instrumental social support was assessed using a single item adapted from the Social Provisions Scale [40] (i.e., “Another person(s) helped you with daily tasks like transportation, studying, or other errands”). Participants were asked to rate the extent to which others provided them with instrumental support that day using a seven-point scale, ranging from 1 (*not at all*) to 7 (*very much*).

#### Emotional social support

*Emotional social support sought.* On a four-point scale ranging from 1 (*I didn’t do this at all*) to 4 (*I did this a lot*), participants rated the extent to which they attempted to seek emotional social support in response to stressors that day using a four-item subscale from the COPE inventory [6] (e.g., “I tried to get emotional support from friends or relatives”). The scale demonstrated a high degree of internal consistency (ω_w_ = .91; ω_B_ = .99).

*Emotional social support received.* Received emotional social support was assessed with a one-item measure which was adapted from the Social Provisions Scale [40] (i.e., “You had interactions with others in which the other person provided you with a sense of emotional security and well-being”). Participants were asked to rate the extent to which others provided them with emotional support that day using a seven-point scale, ranging from 1 (*not at all*) to 7 (*very much*).

#### Eating disorder symptoms

Daily eating disorder symptoms relevant to bulimia nervosa—namely, binge eating and use of inappropriate compensatory behaviours—were assessed via a series of checklist items. Each night, participants were provided with the definition of a binge: “an amount of food that you consider excessive or an amount of food that other people would consider excessive, with an associated loss of control or the feeling of being driven or compelled to keep eating” [41, 42]. They then indicated whether they binged that day (i.e., dummy coded as: 1 = *I binged*; 0 = *I did not binge*). Participants also indicated which, if any, compensatory behaviours they engaged in that day from a list of six: vomiting, laxative use, diuretic use, meal skipping, exercising, and drinking fluids to curb appetite. Days involving any compensatory behaviour were assigned a score of 1, and those that did not were assigned a score of 0.

### Analytic approach

Multilevel models using maximum likelihood estimates were conducted using PROC MIXED in SAS 9.3. Criterion variables were participants’ raw scores across all available days on problem-solving, social support sought for emotional reasons, emotional support received, social support sought for instrumental reasons, and instrumental support received. Given that past simulation studies reveal that samples containing 50 or more units at the between-persons level (i.e., level-2) yield adequate statistical power in multilevel data studies [43], we considered the present sample of 124 participants to be sufficiently powered to test the between-persons hypotheses. To optimize statistical power at the within-persons level, while also minimizing participant burden [44], a 14-day period of data collection was selected.

#### Variable creation

*Level-1.* A predictor variable representing daily self-compassion was calculated by subtracting a participant’s mean self-compassion score over the study period from her self-compassion score on a particular day [45]. Thus, daily self-compassion represented the extent to which a participant’s self-compassion level on a given study day deviated from her average level of self-compassion over the 2-week study period. In addition, a difference self-compassion score was computed to represent the change in a participant’s self-compassion level on a given day (i.e., at time *t*) from the previous day (i.e., at time *t *− 1). Significant positive contributions of daily self-compassion to criteria variables would indicate that, consistent with Hypothesis 2a, participants reported more adaptive coping on days when they were more self-compassionate than usual. A significant positive contribution of difference in self-compassion would indicate that participants coped more adaptively on days when they were more self-compassionate than the day before, consistent with Hypothesis 2b.

In addition to the primary predictor variables, several level-1 covariate variables were created. For Model 1, variables representing daily eating disorder symptoms (i.e., binge eating and use of compensatory behaviours) were created using the dummy-coded procedures outlined above. No centering procedures were applied as these variables had meaningful zero points [46]. For Model 2, a difference score was computed for both binge eating and the use of compensatory behaviours; the purpose of this level-1 covariate was to control for changes in eating disorder symptoms from the preceding day. Additionally, for each criterion variable (e.g., problem-solving) in Model 2, a lag version of the criterion variable (e.g., lagged problem-solving) was computed; this represented a participant’s score on the criterion variable on the previous day (i.e., at time *t *− 1).

*Level-2.* As in prior work (e.g., Kelly and Stephen [19]), trait (i.e., mean) self-compassion was calculated by taking the mean of participants’ self-compassion scores across all available study days. Covariate variables representing participants’ trait (i.e., mean) binge eating and use of inappropriate compensatory behaviours were computed in a similar manner.

#### Multilevel models

Two multilevel models were conducted for each criterion variable, with each model being comprised of fixed effects and a random intercept. Initially, BMI was entered as a level-2 covariate in all models but was subsequently removed due to its nonsignificant contribution across all models; none of the reported findings were affected by the omission of BMI.

*Model 1.* Model 1 tested Hypotheses 1 and 2a that trait self-compassion and daily self-compassion, respectively, would be associated with greater use of adaptive coping behaviours, independent of one’s eating disorder symptoms. In Model 1, self-compassion was entered as a fixed effects within-persons predictor variable (i.e., daily self-compassion; level-1) and between-persons (level-2) predictor variable. Binge eating and use of inappropriate compensatory behaviours were entered as both within-persons and between-persons fixed effect covariate variables. A random intercept was also included in this model.

*Model 2.* Model 2 tested Hypothesis 2b, that increases in self-compassion from the preceding day would be associated with greater use of adaptive coping strategies, when controlling for the preceding day’s level of adaptive coping and changes in eating disorder symptoms from the preceding day. Model 2 was comprised of the following within-persons (level-1) fixed effects: a difference self-compassion score, a difference score for both binge eating and use of inappropriate compensatory behaviours, and a lagged version of the respective criterion variable. A random intercept was also included in the model.

## Results

### Preliminary analyses

Participants responded to an average of 12.43 (*SD* = 2.00) nightly surveys. Within-persons, the mean self-compassion score over the 2 weeks was 2.59 out of 5 (*SD* = 0.59; range = 1.14–3.97) and the mean standard deviation (i.e., the average variability in daily self-compassion scores) was 0.45 (range = 0.06–1.07). The descriptive statistics as well as the intraclass, Pearson (i.e., between-persons), and repeated measures (i.e., within-persons) correlations for all study variables are presented in Table 1.

**Table 1:** Descriptive statistics, intraclass correlations, and between- and within-persons correlations between study variables

	*M*	*SD*	ICC	1	2	3	4	5	6	7	8
1. Self-compassion	2.59	0.59	.563	–	.17	.20*	.28**	.20*	.25**	−.07	.05
2. Problem-solving	1.83	0.38	.373	.15***	–	.52***	.31***	.50***	.38***	−.22*	−.03
3. Instrumental support sought	1.70	0.54	.378	.07**	.24***	–	.61***	.81***	.59***	−.12	.06
4. Instrumental support received	2.77	1.27	.352	.11***	.13***	.32***	–	.64***	.63***	−.04	.03
5. Emotional support sought	1.87	0.57	.355	.09***	.26***	.68***	.31***	–	.76***	−.16	.01
6. Emotional support received	3.25	1.32	.405	.14***	.20***	.48***	.43***	.55***	–	−.06	.005
7. Binge eating	5.31	2.52	.070	−.13***	−.03	.01	−.04	−.03	−.01	–	−.25**
8. Compensatory behaviours	11.12	3.49	.194	−.08**	.001	−.03	−.003	−.02	−.01	−.01	–

Means and standard deviations for all variables were based on mean levels across the 2 weeks. Between-persons correlations are above the diagonal and within-persons correlations are below. Between-persons correlations represent correlations between person-level means and thus reflect the extent to which participants’ mean scores on the different measures over the 2 weeks were related to one another. Within-persons correlations represent the extent to which a participant’s scores on different measures on a given day were related to one another

*p < 0.05; **p < 0.01; ***p < 0.001

### Central analyses

#### Problem-solving

In Model 1, which controlled for trait and daily eating disorder symptoms, trait self-compassion levels were not associated with the use of problem-solving strategies, contrary to Hypothesis 1, but daily self-compassion levels were positively related (see Table 2). That is, consistent with Hypothesis 2a, on days when participants were more self-compassionate than their typical level, they reported using more problem-solving strategies when faced with a stressful situation. When examining the contribution of day-to-day changes in self-compassion in Model 2, results revealed that, consistent with Hypothesis 2b, increases in self-compassion levels from the previous day were associated with a greater use of problem-solving strategies, even when controlling for changes to eating pathology from the previous day and the previous day’s problem-solving strategies (see Table 3).

**Table 2:** Unstandardized regression coefficients (*B*), standard errors, and effect sizes (*R*^2^_β_) for fixed effects from Model 1

Dependent variable	Fixed Effect	*B*	*SE*	*df*	95% CI	*R*^*2*^_*β*_
Problem-solving	Intercept	1.50***	0.21	108	[1.08, 1.91]	
	Within persons predictors					
	Daily self-compassion	0.13***	0.03	1169	[0.08, 0.18]	.02
	Daily binge eating	−0.01	0.03	1169	[−0.06, 0.05]	< .01
	Daily comp. beh.	0.02	0.04	1169	[−0.06, 0.10]	< .01
	Between persons predictors					
	Mean self-compassion	0.10	0.06	108	[−0.01, 0.22]	.03
	Mean binge eating	−0.25	0.18	108	[−0.61, 0.12]	.02
	Mean comp. beh.	0.23	0.17	108	[−0.10, 0.56]	.02
Instrumental social support sought	Intercept	1.27***	0.29	120	[0.70, 1.85]	
	Within persons predictors					
	Daily self-compassion	0.10**	0.03	1391	[0.03, 0.16]	.01
	Daily binge eating	0.02	0.04	1391	[−0.05, 0.10]	< .01
	Daily comp. beh.	−0.05	0.06	1391	[−0.16, 0.06]	< .01
	Between persons predictors					
	Mean self-compassion	0.17*	0.08	120	[0.01, 0.33]	.04
	Mean binge eating	−0.37	0.25	120	[−0.87, 0.12]	.02
	Mean comp. beh.	0.23	0.22	120	[−0.21, 0.67]	.01
Instrumental social support received	Intercept	0.91	0.67	120	[−0.41, 2.22]	.01
	Within persons predictors					
	Daily self-compassion	0.33***	0.09	1392	[0.16, 0.50]	.01
	Daily binge eating	−0.09	0.09	1392	[−0.27, 0.08]	< .01
	Daily comp. beh.	0.06	0.14	1392	[−0.22, 0.33]	< .01
	Between persons predictors					
	Mean self-compassion	0.60**	0.18	120	[0.24, 0.97]	.08
	Mean binge eating	−0.45	0.58	120	[−1.59, 0.69]	.01
	Mean comp. beh.	0.63	0.51	120	[−0.38, 1.64]	.01

Dependent variable	Fixed effect	*B*	*SE*	*df*	95% CI	*R*^*2*^_*β*_
Emotional social support sought	Intercept	1.45***	0.31	120	[0.84, 2.06]	
	Within persons predictors					
	Daily self-compassion	0.12**	0.04	1391	[0.05, 0.20]	.01
	Daily binge eating	−0.02	0.04	1391	[−0.10, 0.06]	< .01
	Daily comp. beh.	−0.02	0.06	1391	[−0.14, 0.10]	< .01
	Between persons predictors					
	Mean self-compassion	0.17*	0.08	120	[0.01, 0.34]	.03
	Mean binge eating	−0.46	0.27	120	[−0.99, 0.06]	.02
	Mean comp. beh.	0.26	0.24	120	[−0.21, 0.72]	.01
Emotional social support received	Intercept	2.02**	0.70	120	[0.63, 3.42]	
	Within persons predictors					
	Daily self-compassion	0.42***	0.08	1391	[0.27, 0.58]	.02
	Daily binge eating	0.04	0.08	1391	[−0.13, 0.21]	< .01
	Daily comp. beh.	0.02	0.13	1391	[−0.24, 0.27]	< .01
	Between persons predictors					
	Mean self-compassion	0.53**	0.19	120	[0.15, 0.92]	.06
	Mean binge eating	−0.63	0.61	120	[−1.83, 0.58]	.01
	Mean comp. beh.	0.10	0.54	120	[−0.96, 1.17]	< .01

*Note. *Comp. beh. = compensatory behaviours

*p < 0.05; **p < 0.01; ***p < 0.001

**Table 3:** Unstandardized regression coefficients (*B*), standard errors, and effect sizes (*R*^2^_β_) for fixed effects from Model 2

Dependent variable	Fixed effect	*B*	*SE*	*df*	95% CI	*R*^*2*^_*β*_
Problem-solving	Intercept	1.68***	0.06	108	[1.56, 1.81]	
	Lagged problem-solving	0.09**	0.03	1015	[0.03, 0.15]	.01
	Difference binge eating	0.002	0.02	1015	[−0.04, 0.04]	< .01
	Difference comp. beh.	0.05	0.03	1015	[−0.01, 0.11]	< .01
	Difference self-compassion	0.11***	0.02	1015	[0.06, 0.15]	.02
Instrumental social support sought	Intercept	1.48***	0.06	123	[1.36, 1.61]	
	Lagged inst. supp. sought	0.13***	0.03	1242	[0.08, 0.18]	.02
	Difference binge eating	0.02	0.03	1242	[−0.03, 0.07]	< .01
	Difference comp. beh.	−0.03	0.04	1242	[−0.11, 0.05]	< .01
	Difference self-compassion	0.06*	0.03	1242	[.002, 0.12]	< .01
Instrumental social support received	Intercept	2.45***	0.12	123	[2.20, 2.70]	
	Lagged inst. supp. received	0.11***	0.03	1243	[0.06, 0.16]	.02
	Difference binge eating	−0.10	0.07	1243	[−0.23, 0.03]	< .01
	Difference comp. beh.	−0.02	0.10	1243	[−0.22, 0.18]	< .01
	Difference self-compassion	0.20**	0.07	1243	[0.05, 0.34]	.01
Emotional social support sought	Intercept	1.67***	0.07	123	[1.53, 1.80]	
	Lagged emot. supp. sought	0.11***	0.03	1243	[0.06, 0.16]	.01
	Difference binge eating	−0.01	0.03	1243	[−0.07, 0.05]	< .01
	Difference comp. beh.	−0.03	0.05	1243	[−0.11, 0.06]	< .01
	Difference self-compassion	0.05	0.03	1243	[−0.01, 0.12]	< .01
Emotional social support received	Intercept	3.04***	0.14	123	[2.77, 3.32]	
	Lagged emot. supp. received	0.07**	0.03	1241	[0.02, 0.12]	.01
	Difference binge eating	0.03	0.06	1241	[−0.09, 0.15]	< .01
	Difference comp. beh.	−0.08	0.10	1241	[−0.27, 0.10]	< .01
	Difference self-compassion	0.21**	0.07	1241	[0.08, 0.35]	.01

*Note. *Comp. beh. = compensatory behaviours, *inst. supp. = instrumental social support,  emot. supp. = emotional social support*

*p < 0.05; **p < 0.01; ***p < 0.001

#### Instrumental social support

Controlling for mean and daily eating disorder symptoms over the 2 weeks, trait self-compassion and daily self-compassion were both positively associated with social support sought and social support received for instrumental reasons in Model 1 (see Table 2). In other words, consistent with Hypothesis 1, individuals who reported higher average levels of self-compassion over the study period reported seeking more instrumental support from others and receiving more instrumental support from others. Further, consistent with Hypothesis 2a, instrumental support sought and received from others was higher on days of higher-than-usual self-compassion. Findings from Model 2 revealed that, as predicted in Hypothesis 2b, increases in self-compassion from one day to the next were associated with greater instrumental social support sought and received (see Table 3). This finding emerged when controlling for the previous day’s instrumental social support sought and received, as well as changes in eating disorder symptoms from the previous day.

#### Emotional social support

When controlling for both trait and daily self-compassion, trait self-compassion was positively associated with social support that was both sought and received for emotional reasons in Model 1 (see Table 2). Thus, consistent with Hypothesis 1, participants with higher average levels of self-compassion reported both seeking and receiving more emotional social support. Daily self-compassion was also positively related to emotional social support sought and received in Model 1. That is, in line with Hypothesis 2a, on days that participants reported higher-than-usual self-compassion levels, they also reported seeking and receiving greater levels of social support for emotional reasons. In Model 2, findings revealed that, consistent with Hypothesis 2b, increases in self-compassion from the preceding day were associated with greater emotional support *received* from others (see Table 3), even when controlling for emotional social support sought and received on the previous day as well as changes in eating disorder symptoms from the previous day. However, contrary to Hypothesis 2b, increases in self-compassion from one day to the next were not associated with emotional social support *sought* from others in Model 2.

## Discussion

Although self-compassion has been consistently linked to reduced eating pathology in people with eating disorders, this was the first study to examine whether self-compassion levels may be associated with indicators of positive mental health in this population. Our 14-day daily diary study of individuals with symptoms of bulimia nervosa revealed that natural fluctuations in self-compassion levels from day to day, and trait or average self-compassion levels over this time period, were generally associated with the use of more adaptive coping strategies. Thus, these findings suggest that by treating themselves with more self-compassion, individuals with eating disorder symptoms may experience more positive mental health.

At the between-persons level, trait levels of self-compassion were positively associated with the seeking and receiving of both instrumental and emotional social support, suggesting that those individuals with symptoms of bulimia nervosa who generally responded more self-compassionately to their distress sought and received more social support. Contrary to past research in non-eating disorder samples [47], trait self-compassion levels were unrelated to the use of problem-solving strategies. It is possible that the divergent finding is related to differences in study population—i.e., clinical versus non-clinical—and/or measurement. We measured trait self-compassion by aggregating daily self-reports of self-compassion rather than cross-sectionally asking people to report on their typical tendencies, as in other studies. Although aggregated self-report measures typically yield more reliable assessments than single administrations of a self-report scale [48], these forms of measurement typically only correlate moderately with one another [49] meaning caution should be taken when comparing our trait-level findings about self-compassion to past research.

At the within-persons level, findings revealed that on days when participants were more self-compassionate than their usual level or than the preceding day’s level, they engaged in more problem-focused coping aimed at directly reducing stressors, namely problem-solving strategies (e.g., planning, organizing) and the seeking and receiving of instrumental (i.e., practical) social support. Similarly, days of higher-than-usual self-compassion were associated with more emotional approach coping strategies aimed at reducing emotional distress adaptively, like seeking and receiving emotional social support from others. In addition, increases in self-compassion from the day before were associated with more emotional support *received* from others but were unrelated to efforts to *seek* emotional social support. Overall, findings at the within-persons level suggest that the day-to-day fluctuations in self-compassion levels that individuals with bulimia nervosa experience are relevant to their capacity to cope adaptively with distress.

It is important to highlight that at both the within-persons and between-persons levels, self-compassion contributed to adaptive coping while controlling for the contributions of participants’ daily and average eating disorder symptoms (i.e., binge eating and inappropriate compensatory behaviours). As such, our findings suggest that self-compassion might contribute to mental health-promoting behaviours independent of how symptomatic individuals are, reinforcing the notion that mental illness and mental health are not mutually exclusive [50].

### Strengths, limitations, and future directions

The present study had several strengths, including the low attrition rate, high ecological validity, and use of repeated measurement. However, there are several limitations that highlight directions for future research. A first limitation of this study lies in the women-only sample. Second, a self-report screening tool screened for bulimic symptoms. Future research should study individuals of other gender identities and incorporate a structured diagnostic interview. Third, effect sizes were small. Nevertheless, recommendations urge against dismissing small effects as trivial when the outcome examined is important [51] as in the case of mental health. Fourth, future research should examine the replicability of the present findings, as well as build on the present work by examining whether self-compassion promotes other markers of mental health in individuals with eating disorders, such as interpersonal well-being. Fifth, while our sample size at the between-persons level was consistent with recommendations from prior research, simulation studies have demonstrated that a sample size of less than 20–40 units at level-1 (i.e., number of study days) can constrain statistical power [52]. As such, future research should consider extending the present study by increasing the number of responses obtained from each participant, such as by increasing the duration of the study.

Sixth, the correlational study design made it impossible to determine whether there was a causal effect of self-compassion on the use of adaptive coping behaviours; thus, future research should employ experimental research designs to determine whether experimentally increasing an individual’s level of self-compassion impacts their subsequent use of adaptive coping strategies. Finally, the present study prompted participants to retrospectively report on their level of self-compassion and use of adaptive coping behaviours at a single timepoint each day. While common in daily diary studies [53], this form of assessment limits our ability to disentangle the temporal associations between self-compassion and coping *within* a given day, as well as increases the risk of participant responses being subject to recall bias [53, 54]. Future research should address these limitations by collecting data multiple times throughout a given day using ecological momentary assessment.

### Contributions and implications

Findings from the present study suggest that there is merit in continuing to investigate whether day-to-day changes in self-compassion levels affect positive mental health in individuals with eating disorders. Taken at face value, the present results add further support to the theory that differences in self-compassion levels, both within a person and between people, are relevant to adaptive coping, a hallmark of positive mental health. Results specifically highlight that this link between self-compassion and adaptive coping is present in people with symptoms of clinical disorders—in this case, eating disorders—an area that had not yet been investigated. Given the well-established link between greater self-compassion and reduced eating pathology, the present results also suggest that future research should examine whether this association can be explained, at least in part, by the use of adaptive coping behaviours. Indeed, it is plausible that self-compassion reduces the need to engage in eating disorder symptoms indirectly by facilitating adaptive methods through which to manage distress, a theory that requires empirical investigation.

Although current treatments incorporate teachings to bolster problem-solving and social skills [55], the present findings, if corroborated by future work, might suggest that an individual’s ability to employ these strategies in their daily life will vary based on their daily level of self-compassion. Therefore, interventions or strategies known to target and increase self-compassion, like compassion-focused therapy (CFT) [56] and self-compassionate letter writing [57], may not only help to reduce eating disorder pathology, as found in prior research [23], but also help individuals with symptoms of bulimia nervosa respond to challenges in their daily life more adaptively and enjoy greater mental health. Furthermore, treatment approaches that focus on the development of self-compassion could be uniquely beneficial for individuals who are not ready or motivated to work directly on changing their eating disorder behaviours but who are interested in improving their quality of life. Indeed, past research found that adults who had not sought treatment for their eating disorder were motivated to engage with, and ended up adhering to, an intervention comprised of self-compassion practices [57].

## Conclusions

The present findings are the first to suggest that self-compassion may promote certain aspects of positive mental health in individuals with eating disorder symptoms. Findings suggest that, independent of how much they are bingeing and purging, being more self-compassionate than others with symptoms of bulimia nervosa, than their typical level, and/or than their preceding day’s level, may promote the use of adaptive coping in individuals with symptoms of bulimia nervosa. While preliminary, the present findings highlight an understudied, yet potentially fruitful direction for future research, namely, exploring whether encouraging individuals with eating disorder symptoms to treat themselves with compassion can have beneficial consequences for their positive mental health.

## Data Availability

The datasets used and/or analysed during the current study are available from the corresponding author on reasonable request.
